# Investigating the Ketogenic Diet As Treatment for Primary Aggressive Brain Cancer: Challenges and Lessons Learned

**DOI:** 10.3389/fnut.2018.00011

**Published:** 2018-02-23

**Authors:** Kenneth A. Schwartz, Mary Noel, Michele Nikolai, Howard T. Chang

**Affiliations:** ^1^Osteopathic Medical Specialties, Colleges of Human and Osteopathic Medicine, Michigan State University, East Lansing, MI, United States; ^2^Department of Nutrition, Sparrow Hospital, Lansing, MI, United States; ^3^Department of Pathology, Sparrow Hospital, Lansing, MI, United States; ^4^Department of Neurology and Ophthalmology, Colleges of Human and Osteopathic Medicine, Michigan State University, East Lansing, MI, United States

**Keywords:** ketogenic diet, glioblastoma, pilot study, lessons learned, blood ketones

## Abstract

Survival of glioblastoma multiforme (GBM) with the current recommended treatment is poor. Reported median survivals are approximately 8–15 months. Based on recent publications from animal models, combining cancer drugs, radiation, and diet-metabolic treatments may be a new route to better survivals. To investigate this possibility, we have begun a clinical trial that has enrolled 15 subjects using a ketogenic diet (KD) as an addition to current standard treatments that include surgery, radiation therapy, and chemotherapy. Of the 15 enrolled, 10 completed the protocol. This perspective describes the challenges and lessons learned during this clinical trial and discusses the critical elements that are essential for investigating treatment with a KD. We also reviewed and compared various types of KDs. We believe that the diet selected should be standardized within individual clinical trials, and more importantly, the patients’ blood should be monitored for glucose and ketones twice daily so that the supervising dietitian can work with the patient and their caregivers to make appropriate changes in the diet. Compliance with the diet is best in highly motivated patients who have excellent home support from a family member or a friend who can help to overcome administrative, physical, and cognition deficiencies associated with the disease. Treatment of GBM using a KD represents a reasonable investigative approach. This perspective summarizes the challenges and lessons learned implementing and continuing KD therapy while the patients are concurrently being treated with radiation and chemotherapy.

## Introduction

During the period between 2008 and 2012, the annual number of patients afflicted with glioblastoma multiforme (GBM), the most aggressive primary brain cancer, in the United States was calculated to be 10,787, and the estimated number of new patients for 2016 is 12,120 (1, 2). Current treatment protocols for treating primary brain cancers utilizes a multidisciplinary coordinated approach usually involving neurosurgery, radiation therapy, and chemotherapy (3, 4). However, the median survival period for GBM patients remains dismal, ranging from 8 to 15 months (5, 6). The addition of temozolomide to radiation therapy prolongs survival slightly less than 3 months (5).

## Rationale: Ketogenic Diet (KD) as Anticancer Therapy

Individual case reports together with studies in rodents suggest that a KD might be a useful adjunct to the current treatment approach. Normal brain cells and tumor cells may differ in their ability to utilize ketones as a metabolic fuel (7, 8). Under physiologic conditions, normal brain cells can obtain energy from either glucose or ketones. In contrast, many tumors become more dependent on glucose for energy support because they have decreased expression of critical ketolytic enzymes (9, 10). Evaluation of enzymatic expression using immunohistochemistry showed that 14 of 17 patients had decreased expression of ketolytic enzymes (10). However, at least in some instances, ketone bodies were detected using proton-magnetic-resonance-spectroscopy in brain tumors of patients who were being treated with a KD (11). It is noteworthy that hyperglycemia is associated with adverse prognosis and postoperative function loss in GBM patients. Patients treated with KD tend to have lower blood sugar levels (12, 13). Another plausible rationale focuses on β-hydroxybutyrate, the main ketone produced during ketosis, which is an endogenous and specific inhibitor of class 1 histone deacetylases capable of activation of specific genes to protect mice against oxidative stress (14). Indeed, the KD affects expression of proteins associated with angiogenesis, invasion potential, and vascular permeability in a mouse glioma model (15). Investigations on seizures and traumatic brain injury showed decreased oxidant production with ketones possibly from improvement in mitochondrial function which could also contribute to anticancer activity (16, 17).

The anti-brain cancer effect of ketones and the KD has been observed in several rodent models (18). Energy-restricted KDs (ERKD) limited the growth of orthotopically transplanted brain tumors in these animals (19). Mice fed ERKD demonstrated higher blood ketone concentrations and reduced brain tumor growth that was associated with an increased rate of apoptosis. KDs also increase the efficacy of metabolic inhibitors for treatment of astrocytomas in rodent models (20). Decrease growth of GBM cell lines was demonstrated in cell cultures treated with β-hydroxybutyrate, the main ketone produced with ketosis (21). In addition, decreased brain tumor growth and increased animal survival was observed in an orthotopic xenograph animal model treated either with a standard KD or a KD supplemented with medium-chain triglycerides (MCT) (21). These reports provide support for investigating the utility of KD in patients with primary brain malignancies.

Ketogenic diets have been suggested as adjuvant cancer therapy and specifically as metabolic therapy for malignant gliomas (22, 23). Our review of the reported cases of glioma patients revealed five patients who responded to treatment with KD (24). Based on these patients and the long and safe history of the use of KDs to treat hard-to-control seizures, we began a pilot protocol to treat primary aggressive brain cancers with a KD (25–27). We agree with the recent review calling for rigorous scientific data to fairly evaluate the role of metabolic treatments for aggressive primary brain cancers (28). The current perspective describes the challenges and lessons learned in the patients enrolled in our trial, under either the initial or the revised protocols.

## Clinical Protocol and Experience with the KD

To date, after signing informed consent 15 patients have been enrolled, and 10 completed treatment with the protocol that was IRB approved (11-452s) and Clinical Trails registered (NCT01535911). Two patients were studied with the original protocol that stipulated starting the KD after they have failed conventional treatments. An additional eight patients have completed the 6 weeks of revised protocol that starts the KD at the same time as the initial radiation and chemotherapy, and continues it for 6 weeks. This protocol has the primary objectives of investigating side effects attributable to the KD, as well as noting tumor response and time to progression. The inclusion criteria are that participants must be over 16 years of age, have histologically confirmed diagnosis of GBM, have an ECOG performance status of ≤2, a life expectancy of >3 months, can tolerate a high-fat diet, and have the ability to give informed consent. Our exclusion criteria are that participants may not have diabetes mellitus, may not have had a cholecystectomy within a year prior to entering the study, do not have any malignancy other than the brain cancer, have not participated in another investigational study within 2 weeks prior to this study’s entrance, do not have brain metastasis from non-brain tumor, do not have any major comorbidity such as liver, kidney, or heart failure, and are not pregnant. The protocol KD is caloric balanced, based on the patient’s starting weight. The KD diet is constructed so that the ratio in grams of fat to combined grams of protein and carbohydrates is 3:1. Before starting and after completing the KD protocol, the patients will have a history and physical exam along with blood for complete blood counts, chemistries lipids, and uric acid. During the 6 weeks of KD, the patient records their daily weight, and twice daily measurements of blood glucose and ketones obtained mornings prior to eating and evenings 2 h after eating. Each patient is given an Omron model HBF-400 scale for their daily weights and an Abbot Precision Xtra Meter with test strips to measure their blood ketones and glucose twice daily. Participants receive dietary instruction by a registered dietitian who develops a meal plan and menus for each subject. In addition, a dietitian calls or visits patients regularly (at least once a week) throughout their time on the KD.

## Protocol Revision, Tolerability, and Side Effects

We revised our protocol based on a report, in a rodent model, that 9 of 11 mice with a transplanted primary brain tumor that were treated with a KD along with radiation therapy survived, whereas all of the control mice and mice treated with just radiation therapy or just the KD died (29). The success of this simultaneous dual treatment in animals prompted a revision of our protocol so that patients’ initial post-surgery treatment includes a KD begun at the same time as radiation therapy and chemotherapy. Eight highly motivated study patients were able to maintain ketosis for 6 weeks with support from their family or caregivers. Participants maintained blood glucoses under 100 mg/dl and blood ketones around 1–2 mM on the average. All participants lost weight, averaging about 5 lbs. Combining the KD with standard-of-care radiation and chemotherapies did not add any significant side effects.

## Comparisons of Different KDs

Our current KD protocol treats patients for 6 weeks, beginning at the same time as they start their radiation therapy and chemotherapy. The diet is calorie balanced, based on the patient’s starting weight, and monitored with twice daily measurements of blood glucose and ketone concentrations to guide diet modifications, if necessary. Because a high-fat KD diet is significantly different and much less palatable than the patients’ usual diet, 6 weeks was as long as our patients could tolerate the KD. If the patient and their family or caregivers are highly motivated, then it is possible to maintain ketosis for 6 weeks.

## KDs Used for Brain Cancers

The diet currently used in our study is calorically balanced and uses a 3:1 ratio in grams of fat to combined grams of carbohydrates and fat. Table 1 includes case reports and clinical trials using KD therapy for patients with brain cancers. Different protocols vary in the timing (at the beginning of treatment, throughout treatment, or after conventional treatments) and the duration of the diet intervention. Most of the reported patients were treated with diet therapy along with at least one other conventional treatment modality. The prescribed calories were not consistently based on body weights, and thus the “calorie load” or “restriction” were not equivalent for all subjects in these studies. Most studies also did not document or log the blood glucose and ketones levels. Thus, it is difficult to know if a particular diet was producing ketosis.

**Table 1:** Human case reports and studies using various ketogenic diet types for patients with brain tumors.

PI	No. of subjects	Diet type	Calorie restriction	Calorie level	Length of study	Type of ketone measurement	Blood glucose measurement	Vitamin/mineral supplement	Commercial nutrition supplements	Treatment phase
**Case reports**										

Nebeling et al. (36)	1	Ketogenic medium chain triglyceride (MCT) oil-based diet (60% MCT oil, 20% protein, 10% carb, 10% dietary fat)	No	85–125% RDA for age; individual-lized to allow for growth	2 months	Blood	Yes	Yes	MCT oil, commercial pediatric formula for 1 subject on tube feedings	Post-chemotherapy for 1 subject, adjunctive with chemotherapy for second subject

Kalamian et al. (37)	1	Atkins × 3 months then Ketogenic 3.5:1 ratio × 9 months	Yes	65–85% RDA	12 months	Blood	Yes	Yes	MCT oil	Adjunctive with Chemotherapy

Zuccoli et al. (38)	1	Ketogenic 4:1 ratio	Yes	600 kcal	2 months	Blood	Yes	Yes	Ketocal, MCT oil	Adjunctive with radiation and chemotherapy

Schwartz et al. (24)	2	Energy-Restricted ketogenic diet (KD): 3:1 ratio	Yes	20–25 kcal/kg/day	6 weeks	Blood	Yes	Yes	No	New diagnosis with radiation and chemotherapy

**PI**	**Study open or closed**	**Diet type**	**Calorie restriction**	**Calorie level**	**Length of study**	**Type of ketone measurement**	**Blood glucose measurement**	**Vitamin/mineral supplement**	**Commercial nutrition supplements**	**Treatment phase**

**Clinical trials**										

Klein et al. (26)	Open	Ketogenic 4:1 ratio	Yes	1,600	6 months	Blood and urine	Yes	Yes	Uses pre-packaged meals	End stage, after radiation and chemotherapy as adjunctive therapy

Klein (39)	Open	Ketogenic 4:1 ratio	Yes	1,600	6 months	Blood	Yes	Not stated	No	New diagnosis with radiation and chemotherapy

Scheck et al. (40)	Open	Ketogenic 4:1 × 6 weeks with radiation/chemo followed by a modified Atkins diet (MAD) during monthly chemotherapy × 12 months	No	Not stated	18 months	Blood	Yes	Not stated	No	4:1 with radiation/chemotherapy, then MAD with chemotherapy

Reiger et al. (41)	Closed	unrestricted KD	<50–60 g carbohydrates/day	Not stated	6 weeks	Urine	No	No	Dietary supplementary products provided by Tavarin	End stage, after radiation and chemotherapy

Schwartz et al. (42)	Open	KD: 3:1 ratio	Yes	20–25 kcal/kg/day	6 weeks	Blood	Yes	Yes	No	New diagnosis with radiation and chemotherapy

## Ketogenic Diets

Ketogenic medical nutrition therapy is not a standardized, scripted diet plan (Table 2). The traditional or classic KD is comprised of a specific fat to carbohydrate (CHO) plus protein (PRO) ratio, fat:(PRO + CHO), usually ranging from a 2:1 ratio (i.e., 2 g of fat to every 1 g of PRO + CHO) to the most strict 4:1 ratio (4 g of fat to every 1 g of PRO + CHO) (30). To achieve these defined ratios, all the macronutrients must be calculated specifically and foods for meals are weighed on a gram scale. Based on maintenance calorie needs, it is unlikely that maintenance protein needs for adults can be met using a 4:1 ratio due to increased body weight. Therefore, 3:1 ratio is typically the highest prescribed for adults and is the diet selected for our protocol. Fat in these diets is provided by a combination of unsaturated and saturated long-chain triglycerides contained in oils like olive, corn, and peanut, and may include MCT oil either as pure supplement or coconut oil which contains MCT oil. These diets, when administered properly, induce measurable ketosis.

**Table 2:** Diet comparison overview.

	Classic ketogenic diet (KD) 4:1 ratio	Classic KD 3:1 ratio	Low Glycemic Index treatment 1.5:1 ratio	MCT oil diet0.67:1–1.5:1 ratio	Modified atkins 0.79:1 ratio	Atkins 0.73:1 ratio
Caloric distribution charts	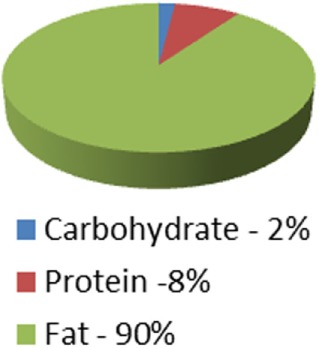	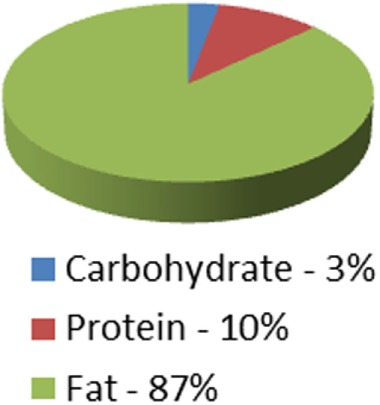	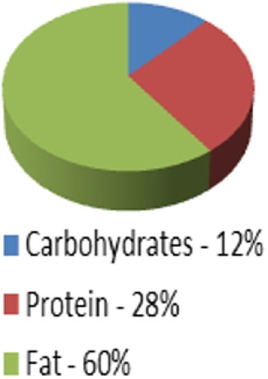	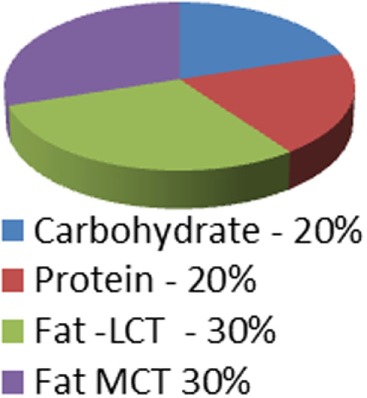	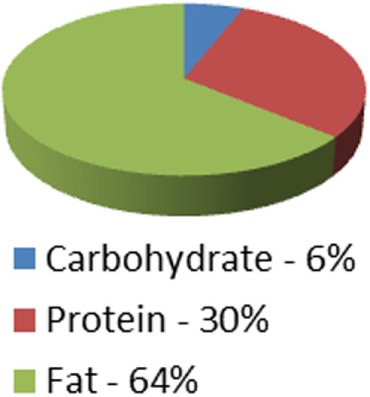	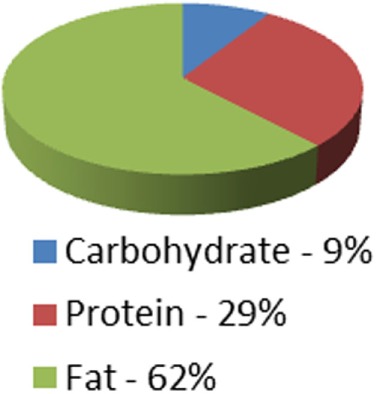

Fat portion of calories	180 g	174 g	120 g	120 g; 60% fat: start with 30% MCT, 30% LCT then may adjust up to 50% MCT^a^ and decrease LCT down to 10% as tolerated	High fat encouraged	High fat encouraged
					Although not measured: At 64% of calories = 128 g	Although not measured: At 62% of calories = 124 g

Protein portion of calories	36 g	45 g	126 g	90 g (can be adjusted up or down)	Although not measured = at 30% of calories = 135 g	Although not measured = at 29% of calories = 131 g

Carbohydrate portion of calories	9 g	15 g	54 g	90 g (can be adjusted up or down)	10–15 g/day for first month	20 g/day for first two weeks
						20 + 5 g/day until 10 lbs from goal weight
					20–30 g/day afterward	+10 g/day until goal weight reached

How are foods measured?	Weighted on digital gram scale	Weighted on digital gram scale	Using household measurements, exchange lists or estimated	Uses exchange lists	Estimated	Estimated

Calories controlled	Yes	Yes	Yes	Yes	No	No

Presence of urine ketones	Yes	Yes	No	Yes^a^ but may need the higher amount of MCT and or ratio adjusted to decrease carb/protein	Yes if protein intake controlled	Yes if protein intake controlled

*^a^Diets adjusted to 1,800 kcal diet for comparison purposes only*.

*LCT, long-chain triglycerides; MCT, medium-chain triglyceride*.

When the MCT Oil diet is used (Table 2), the diet has a lower total fat:(PRO + CHO) ratio than the classic KDs. In this plan, instead of weighing foods, exchange lists based on fat are used to prepare meals. Ketosis can be induced but usually only if the ratio is adjusted to the higher range of MCT oil (up to 50–60% of the fat as MCT oil) (30). The Low Glycemic Index Treatment (LGIT) diet has a fat:(PRO + CHO) ratio similar to the MCT Oil diet, but in the absence of the relatively high amount of MCT oil, usually does not induce ketosis. This diet also uses exchange lists or household measurements for portion control. The LGIT diet has been used in patients with epilepsy with a success rate similar to the classic KD (31). Its usage for people with cancer has not been documented. The Modified Atkins diet and Atkins diet (AD) both have lower fat:(PRO + CHO) ratios than the other KDs, but have a lower proportion of carbohydrate than the MCT Oil and LGIT diets (32, 33). These ADs, similar to the MCT Oil diet and the LGIT diet, do not measure foods specifically, but use household measures to estimate appropriate portions. As long as protein intake is controlled (not too high) and carbohydrate goals are not exceeded, ketosis can be achieved with ADs (34, 35). Diets that do not require weighing specific foods tend to be easier for patients to follow.

## Lessons Learned That Help to Promote KD in GBM Patients

1. Our initial protocol treated patients after they had completed standard-of-care treatments and stipulated that patients had to be hospitalized to teach them about the KD. Our first two patients were hospitalized while being started on the diet. However, after we changed the protocol (to treat patients with the KD after their neurosurgery and beginning at the same time as they were being treated with radiation and chemotherapy), we found that we could initiate the diet as outpatient treatment. All of our subsequent patients have started and maintained the KD without the need for hospitalization.
2. Brain cancer patients frequently have problems in areas like executive functioning, thinking, coordination, and vision that can make instituting and complying with an extremely restricted diet very difficult or almost impossible. Most of our patients required the help of a family member or caregiver to fulfill the KD’s requirements.
3. Since this is a life-threatening illness, it was often difficult for caregivers to restrict calorie intake as well as to forbid foods that patients find comforting, familiar and drawn to in a time of illness.
4. The preparation and palatability of the food can be a challenge to the patient as well as caregiver. It is imperative that patients are instructed carefully, and followed regularly with the supervising dietitian to ensure that ketosis has been achieved and is maintained.
5. Hospitalizations in an acute or chronic care facility (including rehabilitation centers, assisted living, or long-term care facility) can be a problem since many of these institutions may be ill equipped to handle the diet with its strict weighing of the food.
6. Because of the dire prognosis of this diagnosis, patients and their families often looked for all potential alternative treatments that may complicate attempts to investigate the efficacy of the KD as a single variable.
7. The twice daily blood sampling (to measure the ketones and glucose levels) is an added burden to an already stressful situation. However, most of our study patients were able to comply with this protocol stipulation.
8. Because of the strictness and limitations of the KD, socializing with friends and family around a meal can be difficult.
9. Six weeks of compliance with the KD was tolerable by most patients and caregivers.
10. Little is known about the efficacy of the KD in treating cancers in humans. Thus, adherence to the strict diet and the documentations of ketosis are imperative to evaluate fairly whether the KD is an effective treatment.
11. Strong alliance between the research staff, the patients, family caregivers, dietitians, and oncology physicians is necessary for the success in completing this research.
12. In our most recent protocol, the patients are concurrently receiving radiation and chemotherapy. Possible therapy side effects of nausea and other gastric distress symptoms may be exacerbated by the KD. Of the eight patients treated, only 1 patient had nausea and decreased appetite, which were attributed to chemotherapy and radiation therapy by her treating physicians.

## Conclusion

There are both *in vitro* and *in vivo* animal studies to suggest that KD has the potential to augment the treatments currently available for patients with aggressive gliomas. The value of KD to treat humans with these malignancies has yet to be proven in clinical trials. There is currently a lack of standard KD protocols so that comparison of different trials is difficult. Rigorous diet management and objective measures of ketosis are required to fairly evaluate the effectiveness of the KD as therapy for aggressive gliomas.

## Author Contributions

KS wrote the manuscript and edited the tables and figures. MNoel conceptualized the review and helped to prepare the tables and figures. MNikolai helped with writing the manuscript and with the preparation of the tables and figures. HC helped with the preparation of the manuscript. All authors helped, read, edited, and approved the final manuscript.

## Conflict of Interest Statement

The authors declare that the research was conducted in the absence of any commercial or financial relationships that could be construed as a potential conflict of interest.
